# Supplementary material for: The causal relationship between the human gut microbiota and pyogenic arthritis: a Mendelian randomization study
Source: Front Cell Infect Microbiol. 2024 Nov 26;14:1452480. doi: 10.3389/fcimb.2024.1452480 (PMC11629706; doi:10.3389/fcimb.2024.1452480)
Supplement: Supplementary file 1 [file DataSheet1.zip › mendelian test/ebi-a-GCST90027548.csv_leave_one_out.pdf]

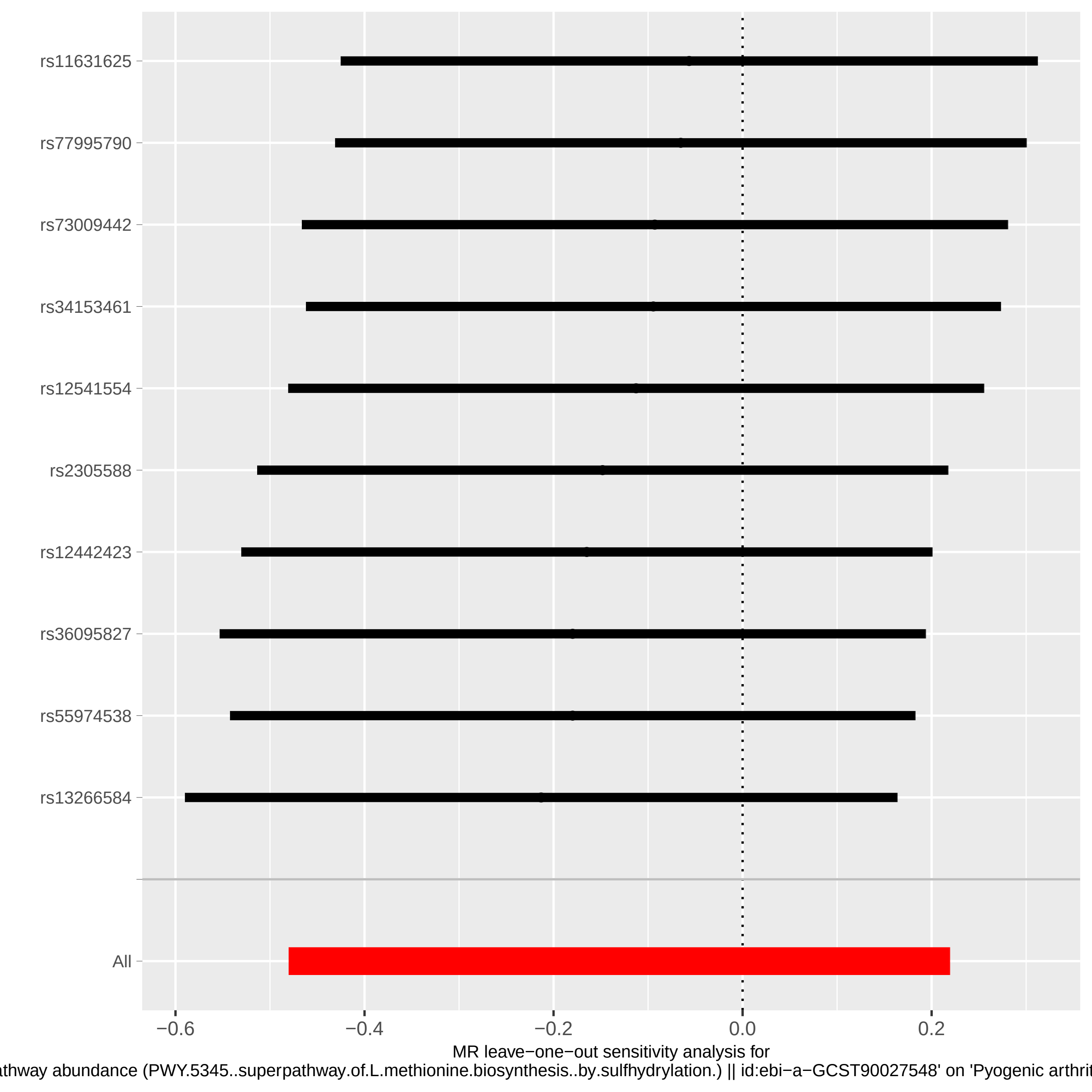
rs11631625
rs77995790
rs73009442
rs34153461
rs12541554
rs2305588
rs12442423
rs36095827
rs55974538
rs13266584
All
−0.6
−0.4
−0.2
0.0
0.2
MR leave−one−out sensitivity analysis for
athway abundance (PWY.5345..superpathway.of.L.methionine.biosynthesis..by.sulfhydrylation.) || id:ebi−a−GCST90027548' on 'Pyogenic arthrit
